# *Nurr1 *regulates *Top IIβ *and functions in axon genesis of mesencephalic dopaminergic neurons

**DOI:** 10.1186/1750-1326-7-4

**Published:** 2012-02-02

**Authors:** Xin Heng, Gang Jin, Xin Zhang, Dehuang Yang, Mingzhe Zhu, Shijun Fu, Xuping Li, Weidong Le

**Affiliations:** 1 Institute of Neurology, Ruijin Hospital, Shanghai Jiao Tong University School of Medicine, Shanghai, 200025, China; 2 Key Laboratory of Stem Cell Biology, Institute of Health Sciences, Shanghai Institutes for Biological Sciences, Chinese Academy of Sciences, Shanghai, 200025, China; 3 Applied Genomics Lab, Institute of Health Sciences, Shanghai Institutes for Biological Sciences, Chinese Academy of Sciences, Shanghai, 200025, China; 4 Department of Neurology, Baylor College of Medicine, Houston, Texas, 77030, USA

## Abstract

**Background:**

NURR1 (also named as NR4A2) is a member of the steroid/thyroid hormone receptor family, which can bind to DNA and modulate expression of target genes. Previous studies have shown that NURR1 is essential for the nigral dopaminergic neuron phenotype and function maintenance, and the defects of the gene are possibly associated with Parkinson's disease (PD).

**Results:**

In this study, we used new born *Nurr1 *knock-out mice combined with Affymetrix genechip technology and real time polymerase chain reaction (PCR) to identify *Nurr1 *regulated genes, which led to the discovery of several transcripts differentially expressed in the nigro-striatal pathway of *Nurr1 *knock-out mice. We found that an axon genesis gene called *Topoisomerase IIβ *(*Top IIβ*) was down-regulated in *Nurr1 *knock-out mice and we identified two functional NURR1 binding sites in the proximal *Top IIβ *promoter. While in *Top IIβ *null mice, we saw a significant loss of dopaminergic neurons in the substantial nigra and lack of neurites along the nigro-striatal pathway. Using specific TOP II antagonist ICRF-193 or *Top IIβ *siRNA in the primary cultures of ventral mesencephalic (VM) neurons, we documented that suppression of TOP IIβ expression resulted in VM neurites shortening and growth cones collapsing. Furthermore, microinjection of ICRF-193 into the mouse medial forebrain bundle (MFB) led to the loss of nigro-striatal projection.

**Conclusion:**

Taken together, our findings suggest that *Top IIβ *might be a down-stream target of *Nurr1*, which might influence the processes of axon genesis in dopaminergic neurons via the regulation of TOP IIβ expression. The *Nurr1-Top IIβ *interaction may shed light on the pathologic role of *Nurr1 *defect in the nigro-striatal pathway deficiency associated with PD.

## Background

Mesencephalic dopaminergic neurons (MDNs) arise from a common set of precursors, but mature to direct a wide range of brain functions [1]. The common feature of these cells is their ability to regulate dopamine (DA) synthesis, transmission and uptake. One of the most important functions MDNs possess is the control over voluntary movements. Also, their degeneration in substantial nigra (SN) is a hallmark of Parkinson's disease (PD) [2]. It becomes a high priority to understand the molecular mechanism and pathway by which MDNs develop and maintain their functions [3,4]. NURR1, a transcription factor belonging to the orphan nuclear receptor superfamily, recognizes DNA by binding hormone-response elements in the promoters of regulated target genes [5]. It regulates the expression of tyrosine hydroxylase (*TH*), dopamine transporter (*DAT*), vesicular monoamine transporter 2 (*VMAT2*), and L-aromatic amino acid decarboxylase (*AADC*), all of which are important in the synthesis and storage of DA [6-9]. *Nurr1 *deficiency results in impaired dopaminergic function and increased vulnerability of MDNs to the oxidative insults [10,11]. Decreased NURR1 expression is found in the autopsied PD midbrains, particularly in neurons containing Lewy bodies, as well as in peripheral lymphocytes of patients with parkinsonian disorders [12]. Also, several studies have found that variations in *Nurr1 *gene might be risk factors for PD [13]. All these studies suggest that NURR1 is essential in the development and differentiation of MDNs phenotype, function maintenance and neuroprotection, and has a distinct role in the pathology of PD [10,14].

Recently, increasing evidence reveals that NURR1 may influence the development and differentiation of MDNs through the regulation of axon genesis. In *Nurr1 *null mice, Wallen and his colleagues, using Fluorogold as a retrograde axonal tracer, did not observe innervations of the striatum by MDN precursors [15]. In primary ventral mesencephalon (VM) cultures, VM cells from wide type (WT) mice showed clear bundles of dopaminergic fibers while VM cells from *Nurr1 *deficient mice displayed a diffuse network of processes without the formation of bundles of dopaminergic fibers after 7 days in culture [16]. An in vitro study also showed that NURR1 induced morphological differentiation in MN9D cells characterized by long, usually bipolar neurites, while mock-transfected cells retained the usual round shape, bearing occasionally very short neurites [17]. It is very interesting to know that gene *neuropilin*, a receptor protein involved in axon guidance and angiogenesis, has been reported as one of *Nurr1 *downstream targets [1,18]. Recently, Jacobs *et al. *performed a study that combined gene expression microarrays and chromatin immunoprecipitation (ChIP)-on-chip analysis on E14.5 *Nurr1*^-/- ^and *Nurr1*^+/+ ^embryos and thereby identified *Dlk1, Ptpru *and *Klhl1 *as novel *Nurr1 *target genes *in vivo *[19]. *Klhl1 *is the homolog of the ACTIN-organizing *kelch *gene in drosophila and is described as playing a central role in neurite outgrowth [20,21]. *PTPRs *have also been involved in axonal growth and guidance [22]. These data raise the possibility that NURR1 may play an important role either directly or indirectly in the fasciculation of dopaminergic axons.

In our efforts to understand the mechanism by which NURR1 regulates dopaminergic cell development and differentiation, we used *Nurr1 *knock-out mice, in combination with microarray technology, to identify novel *Nurr1 *target genes. Using high stringent filtering criteria, several genes were identified as being regulated by *Nurr1*. Of these genes, we are particularly interested in the gene *Topoisomerase IIβ *(*Top IIβ*). Eukaryotic TOP II is present in two isoforms: α and β. The α isoform expresses in proliferating cells and is mainly required for chromosome segregation. The β isoform is enriched in post-mitotic neuronal cells in developing brains [23]. This nuclear enzyme is the catalytic entity operating directly on chromatin DNA and controls and alters the topologic states of DNA during transcription. Previous studies show that TOP IIβ plays a remarkable role in neurodevelopment and axon outgrowth [24-26]. *Top IIβ *knock-out mice exhibit a specific and predominant defect in neuronal development. The defects in motor axon growth in *Top IIβ *mutant mice cause breathing problems and death of the pups shortly after birth [24]. *Nurr1 *knock-out mice also die soon after birth due to respiratory failure [27]. Studies using brain-specific *Top IIβ *knock-out mice have demonstrated an aberrant lamination pattern in the developing cerebral cortex and a similar prenatal death phenotype suggesting an essential role of TOP IIβ in brain development [28]. In the cerebral cortex of *Top IIβ *null mice, neurons born at later stages of corticogenesis fail to migrate to the superficial layers, motor axons fail to contact skeletal muscles, and sensory axons fail to enter the spinal cord. Isolated cortical neurons from *Top IIβ *knock-out embryos exhibit shorter neurites than those from their wild type counterparts, confirming the role of TOP IIβ in neurite outgrowth [26]. TOP II inhibitor ICRF-193 significantly blocks neurite outgrowth and growth cone formation in cultured cerebella granule neurons, dorsal root ganglions and cortical neurons [26].

The present study aims to test the hypothesis that *Top IIβ *is a downstream target of *Nurr1 *and NURR1 might influence the processes of axon genesis via the regulation of TOP IIβ expression.

## Results

### Agenesis of dopaminergic neuron axons in *Nurr1 *knock-out mice and in *Nurr1 *deficient SH-SY5Y cell line

Sagittal sections of postnatal brains of WT, *Nurr1*^+/- ^and *Nurr1*^-/- ^mice were stained with TH antibody. In WT mouse brains, a big bunch of TH^+ ^axons extended rostrally along the medial forebrain bundle (MFB), and then spread to the entire striatal complex. In *Nurr1*^+/- ^mice, we only observed sparse TH^+ ^axons processed along the nigro-striatal pathway. The density of the TH^+ ^nerve terminals in the striatum also declined significantly. In parallel with the total loss of TH^+ ^neurons in SN of *Nurr1*^-/- ^mice, little TH^+ ^axons were observed in the pathway (data not shown).

**Figure 1:**
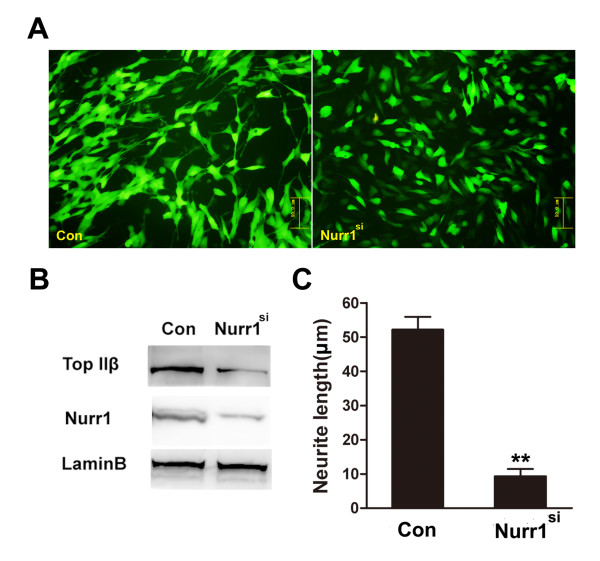
**Axon genesis deficiency in *Nurr1 *deficient SH-SY5Y cell line**. **A**. SH-SY5Y cells were respectively transfected with pSUPER-random and pSUPER-*Nurr1 *siRNA plasmids. After selection by neomycin, stably transfected cell lines with GFP fluorescence were picked on an inverted fluorescence microscope. **B**. The clones with decreased NURR1 expression level were identified by Western blot analysis. TOP IIβ protein level was also analyzed in *Nurr1 *deficient cell line. **C**. The average neurite length (total neurite length/cell number) was determined from more than 100 cells. Bars represent the mean ± SEM (n = 3 independent experiments). Neurite outgrowth in the pSUPER-random and pSUPER-*Nurr1 *siRNA plasmids transfected cell lines showed significant difference. **P <0.01 vs. random control.

To verify the *in vivo *result and study the role of NURR1 on the neurites that have already been extended, stable SH-SY5Y cell lines were screened out after transfection with *Nurr1 *siRNA plasmids or scrambled siRNA plasmids. The SH-SY5Y cell line is a human derived neuroblastoma cell line. The cells both propagate via mitosis and differentiate by extending neurites to the surrounding area. Clones with decreased expression of NURR1 were identified by Western blot analysis. As shown in Figure 1, *Nurr1 *siRNA plasmids inhibited neurite outgrowth of SH-SY5Y cells significantly (about 80% reduction of the neurite length). The *in vitro *data indicate that NURR1 is necessary for the maintenance of extended neurites.

### *Top IIβ *is identified as a target of *Nurr1*

Microarray study in early embryonic stage of *Nurr1*^-/- ^mice has been reported [19]. The purpose of this study was to determine the expression profiles at the late development stage after *Nurr1 *gene was knocked out. According to the study by Wallen et al., even in the absence of *Nurr1*, neuronal differentiation was induced and expression of several genes, including homeobox transcription factors *engrailed *and *Ptx-3 *as well as *aldehyde dehydrogenase 2*, could be detected in developing dopaminergic cells [15]. It means the dopaminergic progenitor cells may still exist in this area. We propose that the loss of TH^+ ^neurons in VM area of *Nurr1*^-/- ^mice may be due to the defect of the terminal differentiation of these progenitor cells. Based on our previous work at our lab using zebra fish model, we found that the dopaminergic neurons still exists in the posterior tuberculum of the zebra fish after we knocked down NURR1 expression level using morpholino technique, suggesting that NURR1 may play an important role in the differentiation and maturation rather than the survival of dopaminergic progenitors in the posterior tuberculum area during zebrafish embryogenesis [29]. Therefore, it is of importance to study the gene expression changes of these non-terminal differentiated dopaminergic progenitor cells. Microarray analysis was employed and several genes were identified with altered expression in the mesencephalon of WT and *Nurr1*^-/- ^mice at P1 (Additional file 1, **Table S1**). Some of these genes have been previously reported to be regulated by *Nurr1*, including *Dat *and *Aldh1a1*, giving confidence that the microarray results represented real changes in expression *in vivo*. We also found that some known *Nurr1 *targeted genes were not in this list. This could be due to our high stringent filtering criteria. For example, *TH*, a known *Nurr1 *regulated gene, is down regulated 1.85 fold in *Nurr1^-/- ^*mice, thus missing the stringent criteria for inclusion. Among the potential targets of *Nurr1, Top IIβ *was highly focused on for its role in axon genesis [24-26]. Quantitative real-time polymerase chain reaction (PCR) analysis of *Top IIβ *also showed a 1.75 fold down regulation in *Nurr1 *^-/- ^mice compared with WT ones.

**Figure 2:**
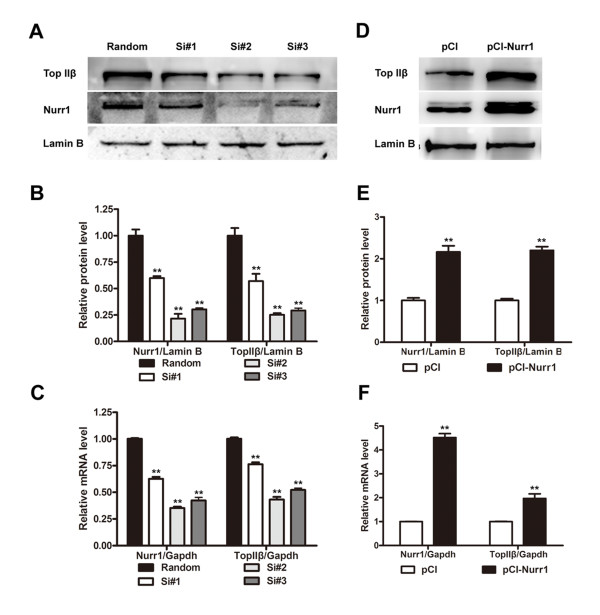
**NURR1 is necessary for the maintenance of TOP IIβ expression in cell cultures**. **A**. Knock-down NURR1 expression level in N2a cells by stealth siRNA down-regulates TOP IIβ protein level. Nucleoprotein of the cells was extracted 3 days after transfection. **B**. Statistic analysis of the protein levels. Data obtained for NURR1 and TOP IIβ expression levels were normalized to LAMIN B expression level. **C**. Knock-down NURR1 expression level in N2a cells by stealth siRNA down-regulates *Top IIβ *mRNA expression. Total RNA of the cells was extracted 48 hours after transfection. We further validated the expression of *Nurr1 *and *Top IIβ *mRNA by real-time PCR analysis. Data obtained for *Nurr1 *and *Top IIβ *expression levels were normalized to *Gapdh *expression level. **D**. Over-expression NURR1 in N2a cells increases TOP IIβ protein level. **E**. Statistic analysis of the protein levels. **F**. Over-expression NURR1 in N2a cells increases *Top IIβ *mRNA expression level. Expression levels expressed relative to a negative control (random sequence). Each bar represents the average of three individual wells, and samples were triplicate. Error bars indicated the standard error for each group. **P <0.01 vs. negative control.

To verify the *in vivo *results, we examined the effect of decreasing or over-expressing NURR1 level on TOP IIβ expression in a cell line that expresses both of the genes under normal growth conditions. The cells chosen were Neuro-2a (N2a) cells, a murine albino neuroblastoma, which constitutes a model system for neurons and starts to be used as an expression system [30]. NURR1 expression was blocked using three sequences of stealth siRNA targeting different sites of mouse *Nurr1 *gene. As shown in Figure 2A-C, application of the three different target sequences significantly suppressed the NURR1 mRNA and protein expression levels, and consequently decreased the TOP IIβ expression levels. When we transiently over-expressed NURR1 in N2a cells, both the mRNA and protein expression levels of TOP IIβ were also elevated (Figure 2D-F). In this study, scrambled siRNA was used as a negative control and LAMIN B was used as an internal control for the nucleoprotein. These data indicate that NURR1 could regulate the expression of TOP IIβ and NURR1 is necessary for the maintenance of TOP IIβ expression in cell cultures.

### The upstream promoter region of the *Top IIβ *gene can be transactivated by NURR1 and contains multiple NGFI-B response element (NBRE)-like sequence motifs

NURR1 was originally characterized by binding as a monomer to a sequence motif named NBRE: 5'-AAAGGTCA-3' [31,32]. To determine whether NURR1 transactivates the *Top IIβ *promoter activity via interacting with binding motif(s), we searched the 5' flanking sequences for the potential NURR1-binding sequence motifs. Remarkably, 11 NBRE-like motifs (no more than one base deviation from the consensus NBRE) were identified within the 10 kb upstream region (Table 1). Among these, two reside within 800 bp proximal region: one at -174 to -167 bp and the other at -720 to -713 bp. A 1600 bp fragment of the mouse genomic sequence upstream of the *Top IIβ *transcription start site (*Top IIβ*+237-1480) containing the two potential NBREs was isolated and cloned into a luciferase reporter plasmid pGL3-Basic.

**Table 1:** Potential NURR1-interacting sequence motifs residing in the 5' upstream flanking region of the *Top IIβ *promoter

Location	Sequence	Orientation
From -174 to -167	TGACgTTT	R
From -720 to -713	AAAGGTCt	F
From -1570 to -1563	cAAGGTCA	F
From -1752 to -1745	TGACtTTT	R
From -2369 to -2362	AAAtGTCA	F
From -3197 to -3190	cAAGGTCA	F
From -4401 to -4394	TtACCTTT	R
From -4444 to -4437	TGACCTTa	R
From -4508 to -4501	aGACCTTT	R
From -6190 to -6183	AAAGGTCA	F
From -7586 to -7579	AAAtGTCA	F
From -9890 to -9883	TGACCTTg	R

The 5' flanking sequences were searched for the presence of NURR1-binding sequence motifs. Thirteen sequence motifs were identified to have no more than one base deviation from the consensus NBRE within the 10 kb upstream region. The deviated base is shown in lower case. The orientation (R: reverse, F: forward) and location relative to the transcription start site of each motif are shown.

**Figure 3:**
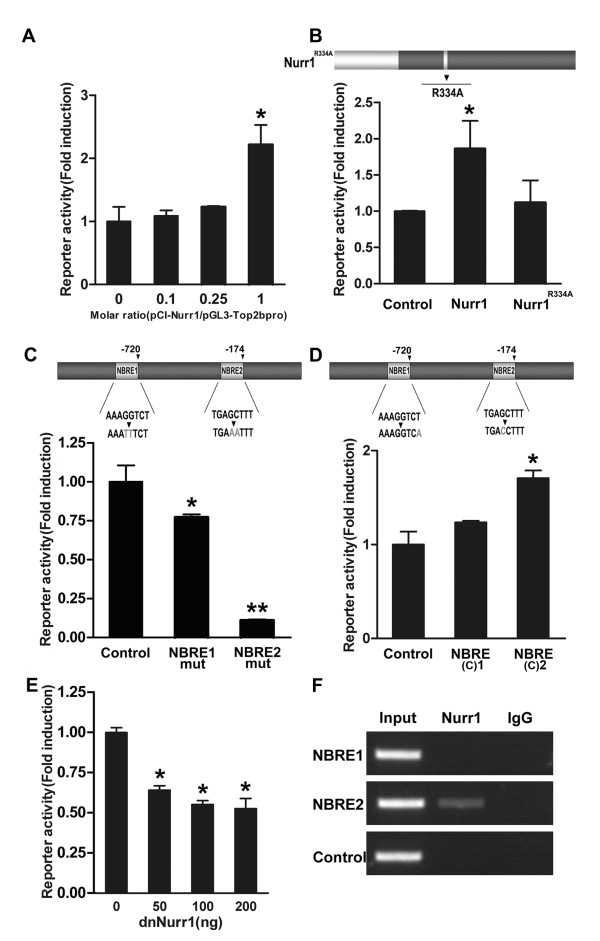
**NURR1 activates the *Top IIβ *promoter through NURR1 binding response elements**. **A**. NURR1 transactivates the *Top IIβ *promoter in a dose-dependent manner in SH-SY5Y cells. The effect plasmid pCI-*Nurr1 *was co-transfected with the reporter plasmid pGL3-*Top IIβ *pro into SH-SY5Y cells. The luciferase activity in the presence of empty vector (pCI) was set to 1. **B**. Structural requirement for activation of the *Top IIβ *promoter. SH-SY5Y cells were transiently transfected with *Nurr1 *mutation construct, which contains a DNA binding deficient mutant (*Nurr1^R334A^*). Data is shown as fold induction relative to cells transfected with empty vector. **C**. Point mutations that disrupt the two putative NBREs clearly reduced the ability of NURR1 to activate the promoter. Data is shown as percentage of the luciferase activity relative to cells transfected with the original promoter construct. **D**. Responsiveness to NURR1 is increased when either NBRE1 or NBRE2 site is converted to a consensus NBRE motif. Data is shown as fold induction relative to cells transfected with the original promoter construct. **E**. Down-regulation of the basal activity of the *Top IIβ *promoter by dnNURR1. SH-SY5Y cells were transiently transfected with the promoter construct together with increasing amounts of dnNURR1 expression plasmids. Data is shown as percentage of the basal activity of the promoter constructs. For the experiments above, the means of relative luciferase activity (induction fold) ± SEM are presented as the average values from three independent experiments. Samples are triplicate. *p < 0.05 and **p < 0.01 vs. control. **F**. ChIP analysis of N2a cells shows recruitment of NURR1 to the endogenous *Top IIβ *promoter. RT-PCR was performed with primers directed towards NBRE1, NBRE2 or a control region located 3700 base pairs downstream of *Top IIβ *transcriptional start site. Only the fragment contains NBRE2 was precipitated by NURR1 antibody.

In transient transfection, the effect plasmid pCI-*Nurr1 *was co-transfected with the reporter plasmid pGL3-*Top IIβ*pro into SH-SY5Y cells. Different amounts of the effecter plasmid were used in co-transfection assay. To compare the fold transactivation by NURR1, the luciferase activity in the presence of empty vector (pCI) was set to 1. Cells were harvested 48 hours after transfection. As shown in Figure 3A, NURR1 can transactivate the *Top IIβ *promoter activity in a dose-dependent manner in SH-SY5Y cells.

Furthermore, SH-SY5Y cells were transiently transfected with expression vector encoding NURR1^R334A^, a DNA binding deficient mutant, and pGL3-*Top IIβ*pro. As shown in Figure 3B, NURR1^R334A ^was incapable of inducing *Top IIβ *expression. This result indicates that the natural structure of NURR1 is necessary to transactivate the expression of *Top IIβ*.

To investigate whether the two putative NBREs are important for NURR1 dependent activation of the *Top IIβ *promoter, point mutations that disrupt these sequences were introduced into the *Top IIβ *promoter constructs by site-directed mutagenesis. SH-SY5Y cells were transiently transfected with constructs harboring mutations either in NBRE1 or NBRE2. Both of the mutations clearly reduced the ability of NURR1 to activate the promoter (Figure 3C). This result strongly supports the idea that NURR1 directly activates *Top IIβ *transcription by interacting with some or both of these cis-elements. Comparing the two sites, mutation of the NBRE2 site reduced the luciferase activity more robustly. One possible reason may be that NBRE2 is closer to the transcription start site and more sensitive to the activation of NURR1.

Consistent with above results, we generated mutant reporter constructs in which the NBRE1 or NBRE2 site was changed to a consensus NBRE motif (F: AAAGGTCA; R: TGACCTTT). When co-transfected with NURR1 expression plasmid to SH-SY5Y cells, transcription activities of the reporter gene were stimulated more in the mutant constructs than in the original ones. Luciferase activities increased 1.23 fold in NBRE(C)1 construct and 1.7 fold in NBRE(C)2 construct (Figure 3D).

A possible explanation for the relatively weak activation by NURR1 over-expression could be that the promoter is already activated due to basal expression of NURR1 in these cells. Indeed, a dominant negative derivative of NURR1 (dnNURR1) that lacks a functional activation domain, repressed the basal activity of the *Top IIβ *promoter indicating that it competes with endogenous NURR1 in the cells (Figure 3E).

### NURR1 binds directly to the endogenous *Top IIβ *promoter

The above results suggest that NURR1 has the potential to regulate the *Top IIβ *promoter and the identified NBREs contribute to the activation. We used ChIP assay to verify that NURR1 was recruited to these sites at the endogenous promoter. Proteins were cross-linked to chromatin and immunoprecipitated with an antibody against NURR1. PCR primers corresponding to either NBRE1 and NBRE2 or a control sequence located 3700 bp downstream of the transcriptional start site were used in these experiments. As shown in Figure 3F, NURR1 was recruited to the NBRE2 in the *Top IIβ *promoter, but neither to NBRE1 nor to the control region.

### NURR1 and TOP IIβ co-express in the VM of mouse

**Figure 4:**
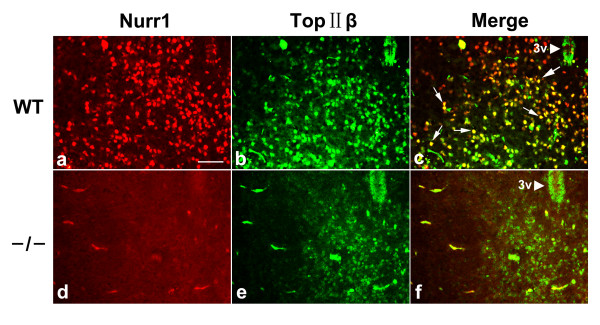
**NURR1 regulates TOP IIβ expression *in vivo***. Both of the proteins located in the nucleus. In newborn WT mice, immunofluorescent analysis showed the co-expression of these two genes in VM area as arrows show (**a-c**). The expression of TOP IIβ in the VM is reduced and limited to medial area in coronal brain sections of *Nurr1 *deficient mice (**d-f**). Arrowheads showed the ventral terminal of the third ventricle (3V). Scale bar = 100 μm.

Next we determined if NURR1 and TOP IIβ co-express *in vivo *in the mouse brain. Expression patterns of NURR1 and TOP IIβ were analyzed by double immunofluorescence staining (Figure 4). Data showed that NURR1 and TOP IIβ co-expressed in nucleus of VM neurons as well as in cells of the hippocampus and cerebrum cortex (Data not shown). TOP IIβ expression seen in the *Nurr1*^-/- ^mice was dramatically weakened and limited to the medial part of this area at P1. Together these findings suggest that NURR1 is essential for high expression of TOP IIβ in the VM area where they co-express.

### TOP IIβ expresses in VM dopaminergic neurons

**Figure 5:**
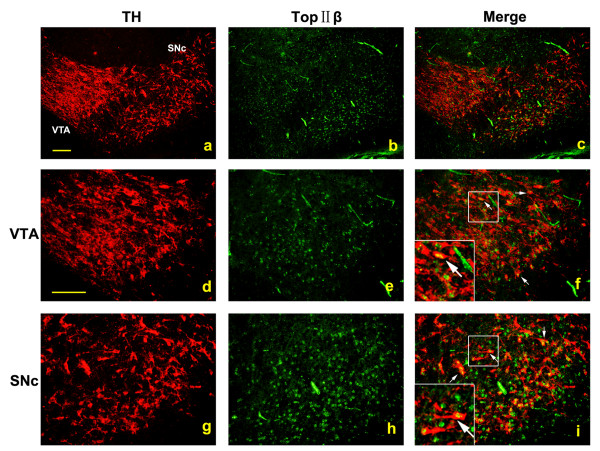
**Co-expression of TH and TOP IIβ in mouse VM**. **a-c **represent an overview of SN and VTA regions. TOP IIβ was found expressed in the nucleus of TH^+ ^neurons in both VTA region (arrows, **d-f**) and SN region (arrows, **g-i**) of P1 mice. Red: TH and green: TOP IIβ. Scale bar = 50 μm.

In order to study the function of TOP IIβ in VM dopaminergic neurons, we designed an experiment to verify whether TOP IIβ expresses in these neurons. Double immunostaining of TH and TOP IIβ was performed in serial coronal sections of the mesencephalons from P1 WT mice. As shown in Figure 5, TOP IIβ localized in the nucleus of TH^+ ^neurons both in SN pars compacta (SNc) and ventral tegmental area (VTA). It indicates that TOP IIβ might be involved in the development of VM dopaminergic neurons.

### TOP IIβ inhibition affects neurite outgrowth and function maintenance of dopaminergic neurons

**Figure 6:**
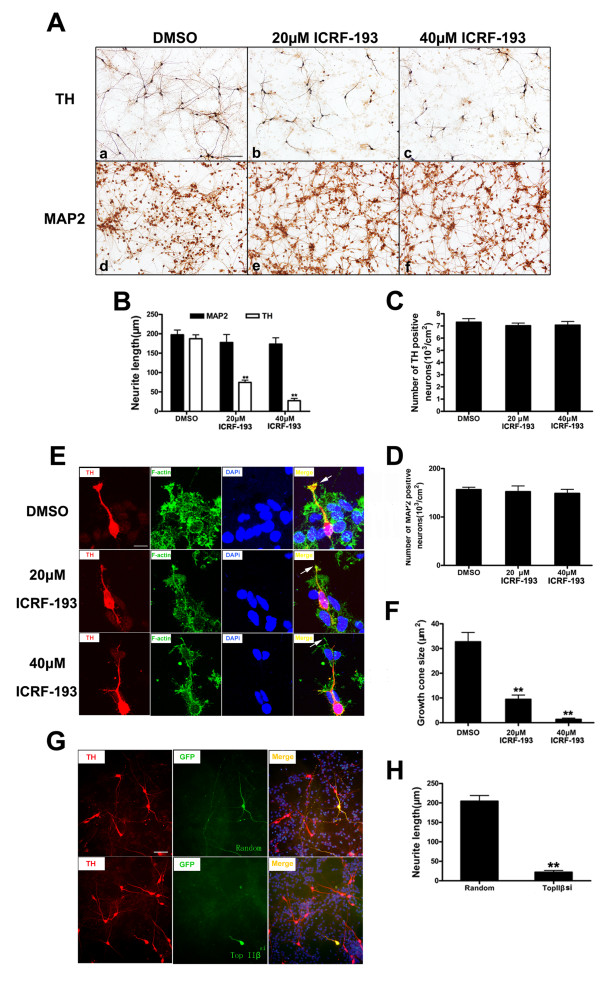
***Top IIβ *deficiency inhibits neurite outgrowth and growth cone formation of primary cultured MDNs**. **A**. ICRF-193 inhibits neurite outgrowth of MDNs. VM neurons isolated and cultured on PDL-coated cover slips. After 2 hours, ICRF-193 was added to a final concentration of 20 μM or 40 μM and the incubation was continued for another 5 days. Neurons were fixed and stained with anti-TH or anti-MAP2 antibodies. Scale bar = 50 μm. **B**. Statistic analysis of neurite lengths. Neurite outgrowth of TH^+ ^neurons in the presence and absence of ICRF-193 showed significant difference, while neurite lengths of the total neurons (MAP2^+^) showed no significant difference. **C**. and **D**. No significant cell number change was found of the total neuron numbers (MAP2^+^) or dopaminergic neuron numbers (TH^+^) after the ICRF-193 treatment by cell counting. **E**. Growth cone formation was abnormal in the presence of ICRF-193. VM neurons isolated and treated with 20 μM, 40 μM ICRF-193 or vehicle for 24 hours. Cells were fixed and stained with Alexa Fluor 488-conjugated phalloidin (for F-ACTIN) and anti-TH antibody. Red: TH, green: F-ACTIN and blue: DAPi. Scale bar = 10 μm. **F**. Statistic analysis of the growth cone size. Area measurement of growth cones was performed by tracing the perimeter of the growth cones using Image J software (NIH, USA). **G**. Neurite outgrowth of MDNs was blocked by *Top IIβ *siRNA plasmids. Primary cultured MDNs were transfected with pSUPER-random or pSUPER-*Top IIβ *siRNA plasmids before seeding using AMAXA nucleofector instrument and cultured for 5 days. Neurons were fixed and stained with anti-TH and anti-GFP antibodies. Red: TH and green: GFP. Scale bar = 50 μm. **H**. Measurement of the neurite lengths of the transfected dopaminergic neurons showed pSUPER-*Top IIβ *siRNA plasmids significantly inhibited the neurite outgrowth. The average neurite length and growth cone size were determined from more than 100 cells. Bars represent the mean ± SEM resulted from 3 independent experiments. **p < 0.01 vs. control.

The catalytic inhibitor of TOP II, ICRF-193, is known to down-regulate TOP II by activating a 26s proteome pathway [33]. The drug binds specifically to the enzyme and stabilizes the TOP II-DNA covalent complex by preventing the ligation of the transiently cleaved DNA strands [34]. VM neurons isolated from 13.5-day-old mouse pups were cultured on PDL-coated cover slips. After 2 hours, ICRF-193 was added to a final concentration of 20 μM or 40 μM and the incubation was continued for another 5 days. Neurons were fixed and stained with anti-MAP2 or anti-TH antibodies. Through determination of the MAP2^+ ^and TH^+ ^neuron loss by cell counting, we found no difference regarding the total neuron number or dopaminergic neuron number between the ICRF-193 and the vehicle treatment groups. However, phenotype-defined dopaminergic neurons exhibited more shortened neurites after ICRF-193 treatment (Figure 6A-D). To confirm the results obtained by the use of ICRF-193, we tranfected the ventral MDNs with *Top IIβ *siRNA plasmids or random sequence control plasmids before seeding using AMAXA nucleofector instrument. Five days after transfection, cells were fixed and stained with anti-TH and anti-GFP antibodies. As showing in Figure 6G and 6H, the dopaminergic neurons transfected with *Top IIβ *siRNA plasmids could not extent their neurites normally, bearing shortened neurites compared with the cells transfected with the random sequence plasmids. These data suggest that the deficiency of *Top IIβ *does not cause the neuron injury in primary VM cultures, but only affects the neurite extension. Dopaminergic neurons seem more vulnerable than general neurons to ICRF-193 exposure.

**Figure 7:**
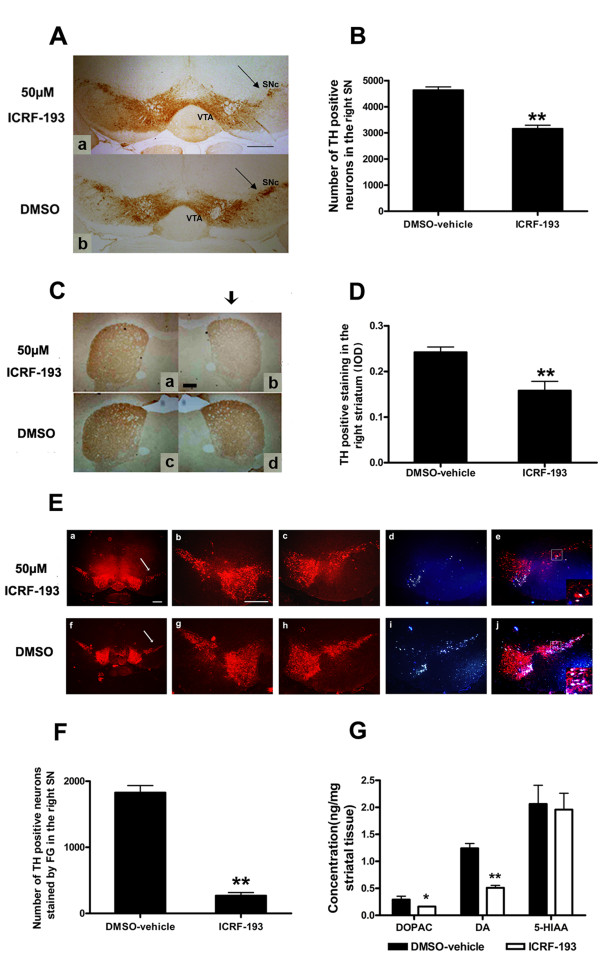
**ICRF-193-mediated TOP IIβ inhibition in SN results in loss of nigro-striatal projection**. **A**. Photomicrographs of TH-immunostained sections of SN of the 2-month-old mice treated with 2 μl 50 μM ICRF-193 (**a**) and vehicle (**b**) for 2 weeks. **C**. Photomicrographs of TH-immunostained sections of striatum of the 2-month-old mice treated with 2 μl ICRF-193 (**a**) and vehicle (**b**) for 2 weeks. **B**. and **D**. Quantitative analysis of the number of TH^+ ^neurons or optical density of striatal TH^+ ^fibers in the right SN (**B**) or striatum (**D**). **E**. Effect of ICRF-193 on nigra-striatum pathway maintenance. SN neurons were labeled by stereotaxic injections of Fluorogold two weeks after ICRF-193 or vehicle treatment. **F**. Statistic analysis showed that the number of Fluorogold labeled dopaminergic neurons was robustly decreased after ICRF-193 injection. **G**. Effect of ICRF-193 on neural transmitter release of dopaminergic neurons. Striatal DA, its metabolite DOPAC and 5-HIAA concentrations were tested by HPLC. The results above are expressed as means ± SEM of 4 mice in each group. *p < 0.05 and **p < 0.01 vs. vehicle control. Arrows show the injection side. Scale bar = 100 μm.

The central component in the road trip of axon genesis is the growth cone, a dynamic structure that is located at the tip of the growing axon. The highly dynamic behavior of the growth cone and its responsiveness to multiple sources of spatial information allows it to find its target with an impressive level of accuracy. The receptors on the surface of the growth cone can sense the guidance cues in the extracellular matrix and the surrounding cell surface and transfer the attractive or repulsive signals into the cell that then guide the neurite to its destination. ICRF-193 treatment affected the growth cone formation in primarily cultured dopaminergic neurons. Morph metric analysis has shown a significant reduction in the size of the growth cones after ICRF-193 treatment compared with vehicle group (Figure 6E and 6F). To determine whether targeting administration of the inhibitor ICRF-193 can induce dopaminergic neuron injury in SN, we stereotaxically injected ICRF-193 into the MFB of C57BL/6 mice. Studies on cytotoxic activity of ICRF-193 in neurons showed that there was not any significant cytotoxic activity of ICRF-193 at or below 100 μM [26]. We found that an injection of 2 μl, 50 μM ICRF-193 into the MFB resulted in a significant loss of nigral TH^+ ^cells and a reduction of optical density of striatal TH^+ ^fibers staining, suggesting that TOP IIβ is necessary for the maintenance of dopaminergic axons in the VM (Figure 7A-D).

### TOP IIβ inhibition *in vivo *affects functional maintenance of nigro-striatum pathway

In order to study the influence on nigro-striatum pathway maintenance of ICRF-193 injection into the MFB, Fluorogold was used as a retrograde tracer. Nigral neurons were labeled by stereotaxic injections of Fluorogold two weeks after ICRF-193 or vehicle treatment. The number of Fluorogold labeled dopaminergic neurons after ICRF-193 injection was markedly decreased, which means the nigro-striatum pathway was injured by ICRF-193 administration and dopaminergic neurons in SN could not extent their neurites to the striatum (Figure 7E and 7F).

We also measured the catecholamine levels in the striatum tissue and found that ICRF-193 injection reduced the levels of DA and its metabolite DOPAC by 43.79% and 58.87% respectively, while 5-HIAA level was not significantly affected (Figure 7G).

### *Top IIβ *deficiency influences the MDNs development and survival in *Top IIβ *knock-out mice

**Figure 8:**
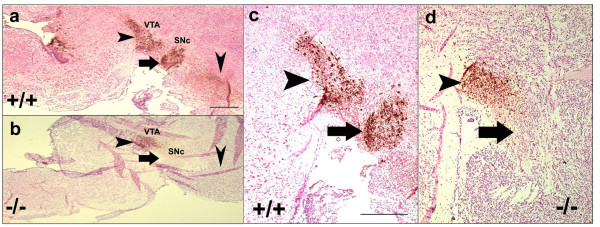
**Loss of TH^+ ^neurons and fibers in the VM of *Top IIβ *null mice**. TH-immunostaining was performed in sagittal sections of E18.5 *Top IIβ*^+/+ ^and *Top IIβ*^-/- ^mice brains. The location of TH^+ ^neurons areas were indicated by horizontal arrows (SNc) and arrowheads (VTA). Vertical arrowheads indicated the location of the TH^+ ^fibers extended from SNc and VTA (**a **and **b**). **c **and **d **showed in higher magnification that the TH^+ ^neurons in SNc was loss in *Top IIβ*^-/- ^mice, while the number also decreased in VTA. Bar = 100 μm.

Expression of TH was dramatically down-regulated in the VM at E18.5 in the *Top IIβ *null mice (Figure 8). We found that the TH^+ ^neurons in SNc were totally lost in the *Top IIβ *null mice and the number of TH^+ ^neurons was also decreased in VTA. In WT mouse brains, we observed a bunch of TH^+ ^axons extended from MDNs rostrally along the MFB, while in *Top IIβ *null mice, we did not detect any fibers generated from the residual TH^+ ^neurons in this area. These findings suggest that TOP IIβ is essential for the development and survival of dopaminergic neurons in the VM and also plays significant roles in the process of axon genesis.

### Over-expression of TOP IIβ rescues *Nurr1 *deficiency-induced neurite impairments in SH-SY5Y cells

**Figure 9:**
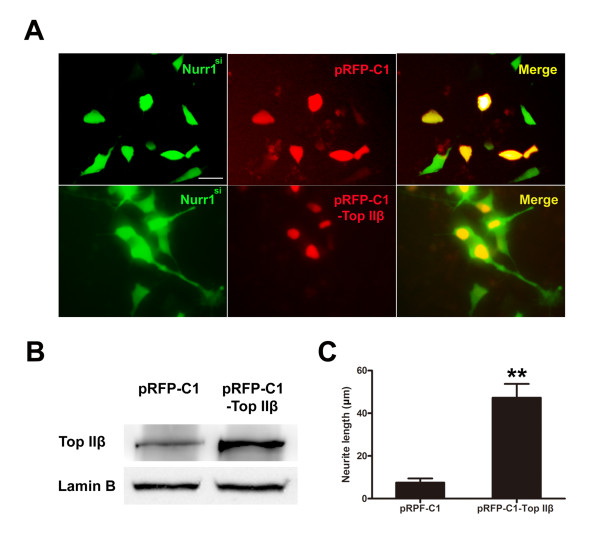
**Over-expression of TOP IIβ rescues *Nurr1 *deficiency-induced neurite impairments in SH-SY5Y cells**. **A**. The pRFP-C1 and pRFP-C1-*Top IIβ *plasmids were transfected to the *Nurr1 *knock-down stable cell line separately. Morphological changes were observed 3 days after transfection. Cells transfected with pRFP-C1 plasmids showed the same morphology as *Nurr1 *deficient cells, bearing occasionally very short neurites. pRFP-C1-*Top IIβ *plasmids express a RFP-fused TOP IIβ protein which located in the nucleus and induced neurites extension. **B**. After transfection for 3 days, pRFP-C1-*Top IIβ *plasmids increased TOP IIβ protein expression level of the cells significantly compared to the empty vectors. **C**. The average neurite length (total neurite length/cell number) was determined from more than 100 cells. Bars represent the mean ± SEM (n = 3 independent experiments). Neurite outgrowth in the pRFP-C1 and pRFP-C1-*Top IIβ *plasmids transfected *Nurr1*-deficiency cell line showed significant difference. **P <0.01 vs. empty vector. Bar = 20 μm.

The pRFP-C1 and pRFP-C1-*Top IIβ *plasmids were transfected to the *Nurr1 *knock-down stable cell line separately. 3 days after transfection, nucleoprotein samples were collected for Western blot assay. pRFP-C1-*Top IIβ *plasmids increased TOP IIβ protein level significantly compared with the empty vectors (Figure 9B). Cells transfected with the plasmids could be identified on an inverted fluorescence microscope (Olympus IX81, Japan). Morphological changes were observed 3 days after transfection. Cells transfected with pRFP-C1 plasmids showed the same morphology as *Nurr1*-deficiency cells, bearing occasionally very short neurites. pRFP-C1-*Top IIβ *plasmids express a RFP-fused TOP IIβ protein, located in the nucleus and inducing neurite extension(Figure 9A). Neurite outgrowth in the pRFP-C1 and pRFP-C1-*Top IIβ *plasmids transfected *Nurr1*-deficiency cell line accounts for a significant difference (Figure 9C).

## Discussion

NURR1 is known to activate transcription and bind DNA as monomer on NBRE that consists of an octanucleotide AAAGGTCA, containing the canonical nuclear receptor hexanucleotide binding motif preceded by two adenines [31,35,36]. According to site-directed mutagenesis and luciferase analysis, we found that both of the NBRE-like sequences located in the 1600 bp *Top IIβ *promoter contributed to the activation of *Top IIβ *transcription although both of them have a one-base deviation from the consensus NBRE. However, NBRE2 is more sensitive to the transactivation of NURR1. One possible explanation is that NBRE2 is located closer to the transcription start site, making it more accessible to other co-activators. As the reporter gene assays totally depend on promoter context with no consideration of the micro-environment of the gene, ChIP assay is performed to determine whether NURR1 is directly recruited to the relevant DNA binding sites in the natural chromatin in living cells. Our data demonstrate that NURR1 is recruited to the NBRE2 in the *Top IIβ *promoter, but not to NBRE1 and the control region located 3700 bp downstream of *Top IIβ *transcriptional start site. It is possible that NURR1 binds weakly to the NBRE1 *in vivo*, and the interaction is undetectable. It is also possible that NURR1 could not bind NBRE1 *in vivo *for the constraints, DNA accessibility, or availability of co-factors in the cell, which strongly dictate where a transcription factor will actually bind. It seems that NBRE2 is the more critical site both for binding in intact chromatin *in vivo *or transcriptional activation *in vitro*.

SN dopaminergic neurons selectively project to dorso-lateral striatum in an orderly medial-to-lateral arrangement, forming the nigro-striatal pathway [37]. Loss of dopaminergic neurons in the SN is one of the main pathological features of PD, which may result from the deficiency of the nigro-striatal pathway. Although the pathway has been identified for more than three decades, the underlying molecular mechanism is not well known. Previous studies suggest that the nigro-striatal circuit is formed via the regulation by many elements such as spatially and temporally distributed guidance cues, neurotrophic factors, morphogens, transcription factors and other known and unknown molecules.

TOP IIβ takes significant role in axon genesis [24], however the precise molecular mechanism is unclear. Ju et al. found evidence that TOP IIβ activates transcription by generating a break in double-stranded DNA within a nucleosome [38]. This enzyme, which is associated with a DNA-repair machinery, allows chromatin to relax, and drive gene expression [38]. Large-scale microarray analysis has revealed that a subset of neuronal genes is down-regulated in the brains of *Top IIβ *knock-out embryos. The expression of genes encoding proteins involved in neuron migration (e.g., *Reln, Dab1, Sst *and *Robo1*), cell adhesion (e.g., *Catna2, Cdh4, Cdh8, Nell2 *and *Alcam*), voltage-gated calcium channel activity (e.g., *Cacna2d1 *and *Cacna2d3*), synaptic transmission (e.g., *Syt1*), and cytoskeleton formation (e.g., *neurofilament*) are down-regulated in the mutant [25]. Using functional immunoprecipitation strategy to identify genomic sites directly targeted by TOP IIβ, several genes were discovered encoding membrane proteins with ion channel, transporter, or receptor activities [38]. Significant proportions of them encode long transcripts and are juxtaposed to a long AT-rich intergenic region [39]. These studies support the notion that TOP IIβ is required for neurite outgrowth during neuronal differentiation, possibly at the level of gene expression.

A recent report also reveals that TOP IIβ is associated with genomic instability in the cerebella region of aging brain [40]. TOP IIβ is treated as an additional biomarker in DNA repair and aging using cultured cerebella granule neurons as an *in vitro *aging model [41]. It has been suggested that neurons may be more sensitive to repair deficiency than other cell types [42]. Thus, the observed neural defects in *Top IIβ *mutant embryos might be related to the plausible involvement of TOP IIβ in DNA repair. Ju et al. identified a connection between initiations of transcription and sensing and repairing of DNA double strand breaks, and found a new chromatin specific function for TOP IIβ [38]. These studies undoubtedly stimulate new conceptual views about the interplay between regulated gene transcription and the DNA damage response.

It has been shown that TOP IIβ is highly expressed in differentiating cerebella neurons. It is the catalytically competent entity operating directly on chromatin DNA *in vivo *[43]. In our studies, we first report the expression profile of TOP IIβ in VM dopaminergic neurons. ICRF-193 is a very significant TOP II poison and causes dose-dependent cross-linking of human TOP IIβ to DNA and stimulates TOP IIβ-mediated DNA cleavage at specific sites on ^32^P-end-labeled DNA [44]. It functions through both TOP IIβ-specific down-regulation and inhibition of TOP IIβ catalytic activity by activating a 26S proteasome pathway [33,45]. TOP IIα-mediated DNA cleavage was stimulated to a lesser extent by ICRF-193 [44]. In the present study, we administrated ICRF-193 in a primary culture of VM neurons as well as nigra-stratum pathway *in vivo*. Our *in vitro *studies indicate that TOP IIβ is required for neurite outgrowth and growth cone formation. However the *in vivo *studies illustrate a significant loss of dopaminergic neurons in SN, striatal dopamine content and injury of the nigra-striatum pathway after stereotaxically injection of ICRF-193 into the MFB of C57BL/6 mice. After TOP IIβ inhibition, the dopaminergic neurons could not maintain the extension of their axons to the striatum, resulting in fail to uptake and retro-transport the neurotrophic factors to the nigral neurons. Consequently, the nigral dopaminergic neurons degenerate and die. In order to rule out the side-effect of ICRF-193, we applied RNAi strategy *in vitro *and observed a similar result as shown in ICRF-193 experiments, confirming that TOP IIβ is required for axon genesis and extension due to its own catalytic function in dopaminergic neurons.

Our results may provide important insights into the mechanisms whereby NURR1 is functioning during mesencephalon development and cell differentiation. Through a combination of *Nurr1 *deficient mice and genomic expression profiling technology, we identified genes, which would possibly mediate the phenotype previously observed in *Nurr1 *deficient mice. Recent studies suggest that many axon-guidance pathway genes, such like *DCC, EPHB1, NTNG1, SEMA5A *and *SLIT3 *were differentially expressed in PD [46,47]. The understanding of the processes of neurite outgrowth, axonal guidance and synaptogenesis is fundamental for developing treatments of PD. The results from our study provide the first evidence and novel molecular mechanisms by which NURR1 interacts with TOP IIβ to regulate MDNs development, differentiation and functional maintenance. These findings may open up a new avenue to explore the possible association of *Nurr1-Top IIβ *in MDNs dysfunction related disease, including PD.

## Conclusion

NURR1 can regulate TOP IIβ expression both *in vivo *and *in vitro. Top IIβ *is a direct downstream target of *Nurr1*. NURR1 can be recruited to the functional NURR1 binding sites which were identified in the *Top IIβ *promoter. The alterations in TOP IIβ are associated with axon genesis in dopaminergic neurons. Knock-down *Top IIβ *affects the neurite outgrowth, growth cone development and nigro-striatum pathway maintenance. *Top IIβ *deficiency seriously influences the MDNs development and survival in developing stages as the MDNs are severely lost in E18.5 *Top IIβ *knock-out mice.

## Methods

### Experimental animals

*Nurr1 *deficient mice were generated by Dr. Conneely's laboratory as previously described [1]. Paired *Nurr1 *knock-out heterozygous (*Nurr1*^+/-^) mice with the same background (129× C57BL/6) were mated to produce the *Nurr1*^+/+^, *Nurr1*^+/- ^and *Nurr1*^-/- ^offspring used in the present study. The genotype of the mice was analyzed with PCR using mouse tail genomic DNA. Three primers were used: a 5' primer (GGCACTCCTGTGTCTAGCTGCC) located in the end of the neo^τ ^gene in exon 3, and two 3' primers, one (CTGCCTTGGGAAAAGCGCCTCC) in the neo^τ ^gene to generate a 200-bp band representing the mutated allele and the other (CAGCCCTCACAAGTGCGAACAC) in a 3' portion of exon 3 to generate a 300-bp WT band [1]. The *Top IIβ*^-/- ^mice were kindly provided by Dr. Yi Lisa Lyu from Department of Pharmacology, UMDNJ-Robert Wood Johnson Medical School [25]. Pups with a *Top IIβ *deletion were identified by the presence of a 450-bp product in a PCR by using the primer pair PR3 (5'-ATATGGTACAGCAACAAAGCATTTGACATA-3') and PR7 (5'-GAATTGTT TGCTGTGGATGCATGTA-3') [28]. WT strains C57BL/6 and ICR were purchased from Experimental Animal Center of Shanghai, China. Mice were mated for 2 hours and subsequently examined for a vaginal plug. Twenty-four hours later, they were designated as E1. Animal care and experimental procedures were performed in accordance with the National Institute of Health Guide for the Care and Use of Laboratory Animals (NIH Publications No. 80-23) and the Laboratory Animal Care Guidelines approved by Shanghai Institutes for Biological Sciences of Chinese Academy of Sciences.

### RNA extraction

Mesencephalic tissues were dissected from newborn mice according to method described before [48]. Tissues in each genotyping group were pooled separately and frozen immediately on dry ice. RNA was isolated from tissue samples using RNeasy mini-columns and treated on the column with DNaseI according to manufacturer's instructions (Qiagen Inc., USA). The quality of total RNA was checked with spectrometer and gel electrophoresis.

### Microarray experiment

The expression of ≈ 36,700 transcripts containing ≈ 12,423 genes and ESTs represented on the Affymetrix Mouse Genome 430 gene chips was quantified in pooled samples from *Nurr1*-null homozygous and wide type mice. For each array, the RNA used was from samples pooled from 5 animals in each group. Triplicate hybridizations were performed for each sample. cDNA synthesis, cRNA labeling, hybridization and scanning were accomplished according to manufacturer's instructions (Affymetrix). The raw data were initially analyzed using Microarray Suite version 5.0 (Affymetrix), which calculates normalized expression levels and generates ratios of experimental/control signals with P values based on the 8-20 different probe pairs that represent each gene on the array. Then, data sets from comparison files were imported into excel (Microsoft) for further comparative analysis. Analysis parameters for gene filtering used by the software were set to values corresponding to high stringency (difference threshold = 100, ratio threshold = 2.0). Affymetrix NetAffx database (http://www.NetAffx.com) was used to annotate probe sets.

### Real-time PCR analysis

Oligonucleotide primers, probes, and reagents (TaqMan universal PCR Master Mix) were purchased from Applied Biosystems. Five genes were selected and verified by TaqMan real-time PCR. The genes and TaqMan probe ID were shown in Additional file 1, **Table S1**. The experimental conditions were followed in accordance with the manufacturer's protocol. Amplification and detection of specific products were performed in the ABI Prism 7900 sequence detection system (Applied Biosystems), and a standard curve and Ct value were obtained.

Real-time PCR for samples from N2a cells was conducted using SYBR Green Real-time PCR Master Mix (Toyobo, Japan) and the primers are listed as follows: *Nurr1*; 5'-CGGCTCTATGGAGATCATCA-3' and 5'-CAATGGAATCAATCCATTCC-3', *Top IIβ*; 5'-GCCCAACTATGATGCTAGAG-3' and 5'- ACAGCATACTGGTTCTGACC-3', *Gapdh*; 5'-TGACCACAGTCCATGCCATC-3' and 5'-GACGGACACATTGGGGGTAG-3'. Samples were triplicate and each sample was performed in 3 wells.

### Stable transfection and selection of *Nurr1 *knock-down SH-SY5Y cell clones

SH-SY5Y cells were bought from Cell Resources Center of Shanghai Institutes for Biological Sciences, Chinese Academy of Sciences. The cells were grown on poly-D-lysine (Sigma, USA) pre-coated dishes in Dulbecco's modified Eagle's medium, (DMEM, GIBCO-Invitrogen, USA) supplemented with 10% fetal bovine serum (heat-inactivated, GIBCO-Invitrogen). The siRNA target sequences are as follows: *Nurr1 *(5'-CCAGAGTTTGTCAAGTTTA-3') and random (5'-GTGGAGCCGAGTTTCTAAATTCCG-3'). SH-SY5Y cells were transfected with plasmids: pSUPER-*Nurr1 *siRNA or pSUPER-random using lipofectamine 2000 (Invitrogen, USA). Cells that have stably incorporated the GFP plasmid into their genomic DNA were selected with 600 μg/ml neomycin (Duchefa, Holland). The clones were expanded and picked on an inverted fluorescence microscope (Olympus IX81, Japan). Total proteins from each clone were collected. Clones with decreased expression of NURR1 were identified by Western blot analysis using anti-NURR1 antibody (Santa Cruz, USA).

### Neurite length analysis

The total neurite length per neuron was determined and calculated as the sum of the lengths of all neurites of a single neuron. The average total neurite length per experimental condition was determined from a sample of at least 100 neurons from random fields.

### N2a cell culture and transient transfection

N2a cells were bought from Cell Resources Center of Shanghai Institutes for Biological Sciences, Chinese Academy of Sciences. The cells were grown in Dulbecco's modified Eagle's medium (DMEM, GIBCO-Invitrogen, USA) supplemented with 10% fetal bovine serum (heat-inactivated, GIBCO-Invitrogen). Cells were maintained at 37°C in an incubator containing 5% CO_2_. The stealth siRNA target sequences are as follows: *Nurr1*si#1 (5'-UAAACUGUCCGUGCGAACCACUUCU-3'), *Nurr1 *si#2 (5'-UCAACAAUGGAAUCAAUCCAUUCCC-3'), *Nurr1 *si#3 (5'-AGAAAUCGGAGCUGUAUUCUCCCG-3') (Cat. No: 152999), and a random sequence used as a negative control (Cat.No:12935300). N2a cells were transfected with stealth siRNA using lipofectamine 2000 (Invitrogen, USA).

### Western blot analysis

Nucleoprotein was isolated from N2a cells (3 days after transfection) using the Proteo JET™ cytoplasmic and nuclear protein extraction kit (Thermo Fisher Scientific, USA). 50 μg of protein from each sample were loaded onto 10% SDS-PAGE and transferred to polyvinylidene difluoride (PVDF). After being blocked for 1 hour in 5% non-fat milk, the PVDF membrane was incubated with primary antibodies overnight at 4°C. The primary antibodies are listed as follows: anti-NURR1 (Santa Cruz, USA), anti-TOP IIβ (Santa Cruz, USA), anti-LAMIN B (Santa Cruz, USA) and anti-β-ACTIN (Sigma, USA). After washing with TBST, samples were incubated with peroxidase-conjugated secondary antibody. The immunoreaction was developed using Super Signal West Dura Extended Duration Substrate (Pierce Biotechnology, USA), and the signal was quantified by measuring optical density of the bands.

### Plasmids construction

The *Top IIβ *promoter fragment was amplified from mouse genomic DNA using the primers:-1380/+237:5'-AAGGCCCGATGATGGACTTGGGAAAGCT-3' and 5'-ATTGGGATCGCG GATGAGGGACGAGGTT-3'. Base substitutions in the NBRE-like motifs on *Top IIβ *promoter were introduced into the promoter sequence using the Muta-direct kit (Beijing SBS Genetech Co., Ltd) according to the manufacturer's instructions. The following oligo nucleotides were used in the mutagenesis procedure: NBRE1 mutant primer; 5'-GCATTGTTGGGAGGAAATTTCTGTAGCCAGAAAAGG-3', NBRE2 mutant primer; 5'-GTTGCTACCCGCAATGAAATTTTCCCCTCGGGTCCCG-3' (underlined base pairs indicate the mutated bases). NBRE(C)1 primer had a consensus NBRE motif at NBRE1 position by changing T at the last position of NBRE1 to A: 5'- GCATTGTTGGGAGGAAAGGTCAGTAGCCAGAAAAGG-3'. NBRE(C)2 primer had a consensus NBRE motif at NBRE2 position by changing G at the fifth position of NBRE2 to C: 5'-GTTGCTACCCGCAATGACCTTTTCCCCTCGGGTCCCG-3'. All promoter constructs were cloned into the pGL3-Basic plasmid (Promega, USA) containing the firefly luciferase gene. Mouse NURR1 over-expression vector pCI*-Nurr1 *was generously donated by Sotirios Tetradis (University of California, Los Angeles). pCI-*Nurr1^R334A ^*has an alanine in position R334 [17]. Dominant negative *Nurr1 *contains a truncated sequence of *Nurr1 *(amino acids 94-365), followed by the sequence of the repressor domain from the Drosophila Engrailed protein [17]. To generate mouse TOP IIβ over-expression vector, total RNA from mouse midbrain at postnatal day 1 was isolated using TRIzol (Invitrogen, USA) and cDNA synthesis was carried out with reverse transcriptase (Toyobo, Japan). The full open reading frame of mouse *Top IIβ *was amplified by PCR with KOD-plus polymerase (Toyobo, Japan) and the following primers: forward 5'- CTCGAGCTATGGCCAAGTCCAGCCTC -3'; reverse 5'- GTCGACTTAATTAAACATTGCA-3'. The fragment was inserted into pRFP-C1 vector from the sites: XhoI and SalI. pRFP-C1 vector is derived from pEGFP-C1 vector and has a red fluorescent protein coding sequence instead of EGFP. The TOP IIβ protein expressed by the plasmid was fused with RFP.

### Luciferase assay

In preliminary experiments, SH-SY5Y cells were used to set the optimal cell density and plasmid concentration for the luciferase assay experiment. The reporter assays were performed in 24-well plates using lipofectamine 2000 reagent (Invitrogen, USA). For the promoter analysis, the plasmids pGL3-*Top IIβ *NBRE1mut, pGL3-*Top IIβ *NBRE2mut, pGL3-*Top IIβ *NBRE(C)1 and pGL3-*Top IIβ *NBRE(C)2 were transfected with NURR1 over-expression plasmid pCI-*Nurr1*. The renilla luciferase vector pRL-SV40 was used as an internal control. Each well was transfected with 400 ng *Top IIβ *promoter plasmid, 400 ng pCI-*Nurr1 *and 8 ng pRL-SV40. Cells were harvested 48 hours after transfection and lysed in Passive Lysis Buffer (Promega, USA). Firefly and renilla luciferase luminescence were measured using the Dual-Luciferase^® ^Reporter Assay System (Promega, USA) according to the manufacturer's instructions. Firefly luminescence was normalized against renilla luminescence for each well, and relative values (fold induction) were calculated by setting the normalized value of the control transfection to 1. The data was performed in triplicate.

### ChIP

Briefly, 10^6 ^SH-SY5Y cells grown in 10 cm cell dishes were treated with 1% formaldehyde solution for 10 min at 37°C to cross-link histones, and resuspended in lysis buffer containing 1% SDS, 10 mM EDTA, 50 mM Tris-HCl, pH 8.1, and then were sonicated to shear DNA. DNA was recovered, and histone-DNA cross-links were reversed in an aliquot, which subsequently was used in PCR reactions to evaluate the amount of DNA present in various groups. The remaining DNA-histone complexes were used in immunoprecipitation reactions utilizing 1 μg of NURR1 specific antibody (E-20, sc-990x, Santa Cruz, USA) or rabbit IgG as a control antibody (Cell Signaling Technology, USA), and a salmon sperm DNA/Protein A-agarose slurry (GE Healthcare, Sweden). Histone-DNA cross-links were reversed, and DNA was recovered and used in PCR reactions utilizing primers that bracket the proximal NBRE-like sites in the *Top IIβ *promoter. The sequences of the primers that encompass the proximal NBRE-like element were 5'-CTGGGTAAAGTTCTTCGG-3' and 5'-GTGCCACCAGTTAGGGA-3'; 5'-CCCGGAATGACTCTTGACA-3' and 5'-CGGCATAACACGGCACA-3'in the proximal mouse *Top IIβ *promoter; and against a control region 3.7 kb downstream of the transcriptional start site 5'-GACAATGCCCTCGCCTTAC-3' and 5'-GCTTTGGATTTGCCTGAA-3'.

### Primary ventral MDNs cultures

Primary ventral mesencephalic (VM) neuron-enriched cultures isolated from E13.5 mouse fetuses were cultured on PDL-coated coverslips (Wanner Instruments, USA). The cells were allowed to grow in a serum-free medium with B27 (GIBCO-Invitrogen, USA). After 2 hours, ICRF-193 was added to a final concentration of 20 μM or 40 μM separately. The incubation was continued for another 24 hours for growth corn formation analysis and 5 days for neurite length measurement. Cells were fixed and stained with anti-TH antibody and Alexa Fluor 488-conjugated phalloidin for F-ACTIN staining. The neurite length and growth cone area of the immuno-reactive neurons were measured and analyzed. For electroporation, the primary VM neurons were homogenously resuspended with the transfection buffer to a final concentration of 4-5 × 10^6 ^/100 μl and mixed with 2 μg plasmid DNA of either pSUPER- *Top IIβ *siRNA or pSUPER-random. The siRNA target sequences are as follows: *Top IIβ *(5'-CAACTATGATGCTAGAGAA-3') and random (5'-GTGGAGCCGAGTTTCTAAATTCCG -3'). Electroporation was performed with an AMAXA Nucleofector instrument as per the manufacturer's protocol. The transfected neurons were then seeded to 24-well plates containing poly-D-lysine coated coverslips. After 5 days, the cells were fixed and stained with anti-TH and anti-GFP antibodies.

### Immunohistochemical and immnunofluorescent staining

VM cells or tissue sections were fixed in 4% paraformaldehyde at room temperature for 30 min. For immunohistochemical staining, cells or tissue sections were treated with 0.3% H_2_O_2 _and then blocked with a pretreatment solution (PBS containing 4% horse serum and 0.3%Triton-X-100) at room temperature for 30 min, followed by incubation overnight at 4°C with TH antibody (1:500, Millipore, USA). After incubation with biotinylated secondary antibody (1:200, Vector, Burlingame, CA) at room temperature for 2 hours, the Vector ABC kit (Vector, USA) and diaminobenzydine (DAB)-H_2_O_2 _were used to visualize perspective cells. For immnunofluorescent staining, the cells were blocked in the pretreatment solution at room temperature for 30 min, followed by incubation with TH antibody (1:500, Millipore, USA), NURR1 antibody (1:100, Santa Cruz, USA) or TOP IIβ antibody (1:50, Santa Cruz, USA) overnight at 4°C, then with FITC-conjugated (1:400, Vector, USA) or TRITC-conjugated IgG (1:800, Vector, USA) at room temperature for 2 hours. Finally, the visual area was covered with a coverslip mounted with anti-fade Aqua Poly/Mount (Polysciences, USA), and then visualized and photographed using an inverted fluorescence microscope (Olympus IX81, Japan) equipped with a DP70 CCD digital camera (Olympus, Japan).

### Stereotaxic injection

For administration of TOP II inhibitor ICRF-193 *in vivo*, male C57BL/6 mice weighting 22-26 g were anesthetized with chloral hydrate and mounted on a Benchmarker stereotaxic apparatus (myNeurolab, St. Louis, MO, USA) in a sterilized chamber. Each animal received an injection of either ICRF-193 of different concentrations (Biomol, USA) in 2 μl vehicle (0.1% dimethyl sulfoxide, DMSO) or vehicle alone as a control into the right medial forebrain bundle (MFB) at a flow rate of 1 μl/min (AP -1.3 mm, ML ± 1.1 mm DV -5.25 mm from Bregma) [49].

In order to study the influence on nigra-striatum pathway formation of ICRF-193 injection, Fluorogold (Fluorochrome, USA) was used as a retrograde tracer. Two weeks after ICRF-193 or vehicle injection, each animal received an injection of 0.2 μl Fluorogold (AP 0.8 mm, ML ± 2 mm DV -3 mm from Bregma) [49].

### Biochemical analysis of catecholamines

The whole right striatum was dissected and homogenized (10%wt/vol) by sonication in ice-cold 0.2 M perchloric acid with 3, 4-dihydroxybenzylamine (DHBA) as internal standard. Homogenate was centrifuged at 20,000 g for 15 min at 4°C and the supernatant was collected. The levels of DA, 3, 4-dihydroxyphenylacetic acid (DOPAC) and 5-hydroxyindolacetic acid (5-HIAA) were determined by high-pressure liquid chromatography (HPLC; EPC-300, Eicom, Japan) equipped with a column of 5 μm spherical C18 particles and detected with an electrochemical detector. The mobile phase (previously filtered and degassed) consisted of 0.042 M citric acid monohydrate, 0.038 M sodium acetate trihydrate, 0.94 mM sodium octane sulfonate and 0.013 mM EDTA-2Na (pH 3.8).

### Statistics

Statistical significance between groups was assessed by *t*-tests using GraphPad Prism (GraphPad Software, Inc., San Diego, CA). A P-value < 0.05 was considered significant.

## Abbreviations

ChIP: chromatin immunoprecipitation; dominant negative NURR1: dnNURR1; DA: dopamine; DAT: dopamine transporter; MDNs: mesencephalic dopaminergic neurons; MFB: medial forebrain bundle; N2a: Neuro-2a; NBRE: NGFI-B response element; PD: Parkinson's disease; PCR: polymerase chain reaction; SNc: substantial nigra pars compacta; TOP IIβ: Topoisomerase IIβ; TH: tyrosine hydroxylase; VM: ventral mesencephalic; VTA: ventral tegmental area; WT: wide type.

## Competing interests

The authors declare that they have no competing interests.

## Authors' contributions

XH carried out the molecular genetic studies, cell culture, stereotaxic injection, immunohistochemistry and drafted the manuscript. GJ conceived and designed the microarray experiments and provided intellectual input. XZ conceived of the study, and participated in its design and coordination and helped to draft the manuscript. DHY participated in the design of the study and performed the statistic analysis. MZZ and SJF carried out the microarray experiments and performed the data analysis. XPL carried out the TH staining of *Top IIβ *null mice and participated in the design of the study. WDL conceived and designed the experiments and provided intellectual input. All authors read and approved the final manuscript.

## Supplementary Material

Additional file 1 **Comparison of genes expression in *Nurr1*^-/- ^mice vs. WT mice**. Microarray analysis was employed and several genes were identified with altered expression in the mesencephalon of WT and *Nurr1*^-/- ^mice at P1. Click here for file
