# Supplementary material for: Nurr1 regulates Top IIβ and functions in axon genesis of mesencephalic dopaminergic neurons
Source: Mol Neurodegener. 2012 Feb 2;7:4. doi: 10.1186/1750-1326-7-4 (PMC3359158; doi:10.1186/1750-1326-7-4)
Supplement: Additional file 1 — Comparison of genes expression in Nurr1-/- mice vs. WT mice. Microarray analysis was employed and several genes were identified with altered expression in the mesencephalon of WT and Nurr1-/- mice at P1. [file 1750-1326-7-4-S1.DOC]

**Additional file**

**Table S1. Comparison of genes expression in *Nurr1*^-/-^ mice vs. wild type mice**

| **Probe sets** | **Gene Symbol** | 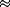**Fold Change in *Nurr1* ^-/-^ mice** | 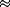**Fold change in *Nurr1*^-/-^ mice by QPCR** |  |
| --- | --- | --- | --- | --- |
| **metabolism** |  |  |  | |
| 1416468_at | *Aldh1a1* | -3.26 | -2.84534 | |
| 1417415_at | *Dat* | -2.04 | -3.53949 | |
| 1459737_s_at | *Ttr* | -9.03 |  | |
| 1423859_a_at | *Ptgds* | -3.4 |  | |
| 1423860_at | *Ptgds* | -3.59 |  | |
| **Axon genesis/guidance** |  |  |  | |
| 1448458_at | *Top IIβ* | -2 | -1.75404 | |
| 1441246_s_at | *Dpysl5* | -2.11 | -2.11443 | |
| **Miscellaneous** |  |  |  | |
| 1438549_a_at | *Srr* | -2.4 |  | |
| 1438562_a_at | *Ptpn2* | 2.25 |  | |
| 1438826_x_at | *Calm3* | -2.24 |  | |
| 1418357_at | *Foxg1* | 3.03 |  | |
| 1421917_at | *Pdgfra* | 2 |  | |
| 1450243_a_at | *Dscr1l1* | -2.04 |  | |
| **Unknown function** |  | |  | |
| 1442680_at |  | 2.12 | 1.75 | |
| 1447982_at | *1110008P14Rik* | 3.74 |  | |
| 1448194_a_at | *H19* | -2.54 |  | |
| 1434147_at |  | -2.01 |  | |
| 1443830_x_at | *Rnf103* | 2.28 |  | |
| 1425521_at | *Paip1* | 2.18 |  | |
| 1434416_a_at | *Solh* | 2.18 |  | |
| 1417714_x_at | *Hba-a1* | -2.11 |  | |

*P*<0.05
